# Growth, maturity, and diet of the pearl whipray (*Fontitrygon margaritella*) from the Bijagós Archipelago, Guinea-Bissau

**DOI:** 10.7717/peerj.12894

**Published:** 2022-03-07

**Authors:** Owen N. Clements, Guido Leurs, Rob Witbaard, Ido Pen, Yvonne I. Verkuil, Laura L. Govers

**Affiliations:** 1 Groningen Institute for Evolutionary Life Sciences (GELIFES), Conservation Ecology Group, University of Groningen, Groningen, The Netherlands; 2 Department of Coastal Systems, Royal Netherlands Institute for Sea Research (NIOZ), Texel, The Netherlands; 3 Department of Estuarine & Delta Systems, Royal Netherlands Institute for Sea Research (NIOZ), Yerseke, The Netherlands; 4 Groningen Institute for Evolutionary Life Sciences (GELIFES), Theoretical Research in Evolutionary Life Sciences, University of Groningen, Groningen, The Netherlands

**Keywords:** Batoidea, Coastal ecology, Life-history, Ontogenetic shifts, Size-at-maturity, Trophic ecology

## Abstract

The pearl whipray *Fontitrygon margaritella* (Compagno & Roberts, 1984) is a common elasmobranch in coastal western African waters. However, knowledge on their life-history and trophic ecology remains limited. Therefore, we aimed to determine the growth, maturity and diet of *F. margaritella* from the Bijagós Archipelago in Guinea-Bissau. Growth was modelled with: von Bertalanffy, Gompertz and logistic functions. Model selection revealed no model significantly outperformed another. The sampled age ranged from less than 1 to 7 years (1.8 ± 1.9 cm, mean ± standard deviation) and size (disc width) ranged from 12.2 to 30.6 cm (18.7 ± 5.2 cm). Size-at-maturity was estimated at 20.3 cm (95% CI [18.8–21.8 cm]) for males and 24.3 cm for females (95% CI [21.9–26.5 cm]), corresponding ages of 2.2 and 3.9 years. The diet differed significantly among young-of-the-year (YOY), juveniles and adults (*p* = 0.001). Diet of all life stages consisted mainly of crustaceans (27.4%, 28.5%, 33.3%) and polychaetes (12.5%, 26.7%, 20.3%), for YOY, juveniles and adults respectively. This study shows that *F. margaritella* is relatively fast-growing, matures early and experiences ontogenetic diet shifts. These results contribute to status assessments and conservation efforts of *F. margaritella* and closely related species.

## Introduction

The abundance of sharks and rays (Elasmobranchs) are indicators of healthy ecosystems, as these species fill important ecological roles as top- and meso-predators. Their population trends may indicate overexploitation of these species, which can potentially alter ecosystem functioning (Barría, Coll & Navarro, 2015; Flowers, Heithaus & Papastamatiou, 2020; Navia, Mejía-Falla & Giraldo, 2007; Vaudo & Heithaus, 2011). Determining such population trends requires information about the life-history of a species, such as the age-at-maturity, maximum age and growth coefficients (Mejía-Falla et al., 2014). A lack of knowledge on life-history parameters can impair status assessment of elasmobranch species, hampering effective management of these K-selected species (*i.e*., late maturity, low fecundity and slow growth) (Ismen, 2003; O’Shea et al., 2013). Furthermore, understanding the trophic ecology of a species is required to determine a species’ ecological role within an ecosystem (Vaudo & Heithaus, 2011). The trophic ecology of a species can help to determine structuring roles, energy flow and bioaccumulation of ecological contaminants within an ecosystem (Bowes & Thorp, 2015; MacNeil, Skomal & Fisk, 2005). Thus, understanding the life-history and trophic ecology of individual species are essential steps in preserving ecosystem functions and services (Coll, Navarro & Palomera, 2013).

Elasmobranch species off the West African coast remain largely unstudied with the necessary data required for population trend analysis and conservation status often missing. This is especially the case for endemic species in the region, like *F. margaritella*. Although this species is one of the most common species in coastal fisheries throughout the region, its life-history characteristics and trophic ecology remain poorly understood (Moore, Séret & Armstrong, 2019). This species ranges from Mauritania to Angola and it can be found in shallow marine and estuarine soft-bottom habitats (Marshall & Cronin, 2016). The maximum attained size is thought to be around 34 cm, and females can have up to three pups per litter (Moore, Séret & Armstrong, 2019). Understanding the life-history and trophic ecology of *F. margaritella* may also provide wider insights into the biology of other *Fontitrygon-*species which mostly occur in data deficient regions off West Africa and the northern coast of South America. Here we specifically aim to fill an important knowledge gap surrounding this species by determining the growth, size and age-at-maturity, and the diet of *F. margaritella* from the Bijagós Archipelago in Guinea Bissau.

## Materials and Methods

**Figure 1:**
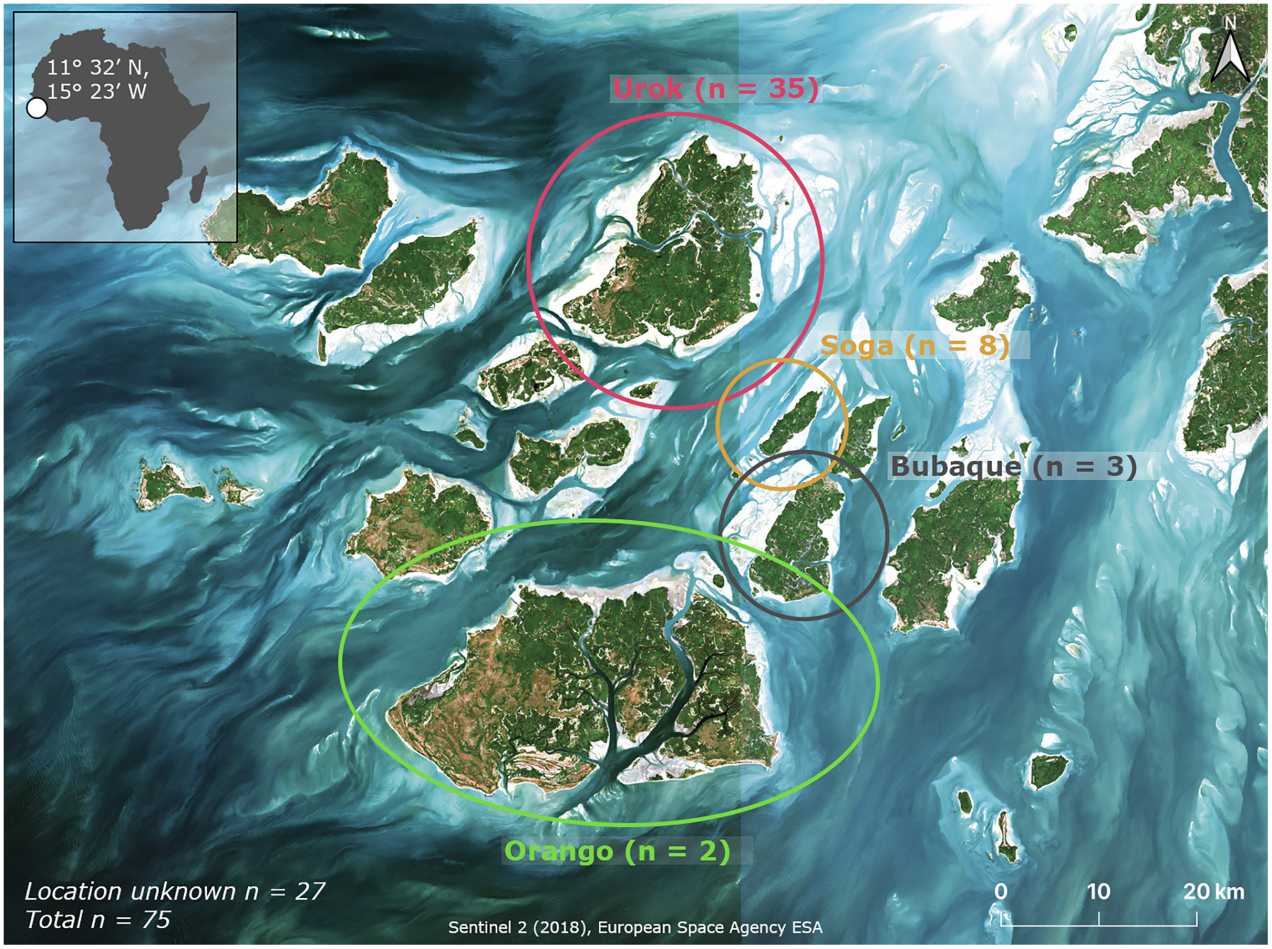
Overview of the study sites in the Bijagós Archipelago, Guinea-Bissau. The colours indicate the different sampling regions and their respective sample sizes (purple = Urok, orange = Soga, grey = Bubaque, and green = Orango). Specimen for which the origin within the archipelago could not be confirmed were labelled as ‘location unknown’.

The Bijagós Archipelago consists of 88 islands and islets and is located off the coast of Guinea Bissau. The archipelago is listed as a UNESCO Biosphere Reserve and as a RAMSAR-site. The coastal zone of the archipelago consists of mangrove forests, soft-bottom intertidal flats, gullies and deep channels. We collected ray specimens between October and December 2019 from artisanal fishers and were caught around Urok (11.4833°N, 15.9667°W), Bubaque (11.2448°N, 15.8701°W), Soga (11.3500°N, 15.8667°W) and Orango (11.2494°N, 162212°W) or from an unknown location within the Bijagós Archipelago (Figure 1). All rays were stored in a field freezer (–10 °C) until processing. To rule out any misidentification of this species with the related daisy whipray (*Fontitrygon margarita*), we sequenced tissue samples of the pelvic fins for species identification. This was done using the ASNM and ChimeraF primer (“AAGGACTACTTTGATAGAGT” a variant of ILEM) adapted from Naylor et al. (2012).

### Ethical statement

For the purpose of this study, we collaborated with the local fishing communities within the Bijagos Archipelago. All rays were obtained from catches by local fishers and were solely captured for consumption purposes. After required samples were collected, all rays were subsequently given back to the local communities for consumption. All rays were deceased at the time of sampling. All research and use of animals was conducted with permission and in accordance with the regulations of the Instituto da Biodiversidade e das Áreas Protegidas (IBAP), the responsible national institute within in Guinea-Bissau (reference number: 396/IBAP/2019 and 393/IBAP/2019).

### Age and growth

For each individual that was sampled, we recorded sex, body size as disc width $\left( {DW} \right)$ and total length $\left( {TL} \right)$ and weight. In addition, five to ten anterior vertebrae were stored in 70% ethanol for each individual. In the laboratory, vertebrae were cleaned by removing excess tissue after which one vertebra per individual was fixated in clear epoxy resin (Poly-Pox THX 500 resin and Poly-Pox 155 hardener) following the instructions of Campana (2014). A centered sagittal cross-section with a thickness of 500 µm was cut for each vertebra, to create a typical ‘bowtie’ cross-section which was fixated to a microscope slide and used for ageing (see Campana, 2014). Each cross-section was photographed using a compound light trinocular microscope (Zeiss) using a 5 × 10 magnification. As growth band deposition in other dasyatid rays like the blue stingray (*Dasyatis chrysonota chrysonota*), the brown stingray (*Dasyatis lata*) and the diamond stingray (*Dasyatis dipterura*) is annual, we assumed deposition in *F. margaritella* also to be annual (Cowley, 1997; Dale & Holland, 2012; Smith, Cailliet & Melendez, 2007). Age was determined independently by two researchers by counting growth bands. If age the first readings a second reading was made, all age reading that differed were taken out of the analysis. Previously, the use of a multi-model approach for growth studies has been advocated to incorporate candidate models with alternative characteristics (Smart et al., 2016). Hence, the following three growth functions were fitted.

A logistic growth function, adapted from McKendrick & Kesava (1912):

(1) $$D{W_{age}} = {\rm \; }\displaystyle{{D{W_{inf}}} \over {1 + \left( {\displaystyle{{D{W_{inf}} - D{W_{birth}}} \over {D{W_{birth}}}}} \right){e^{ - k\ \times\ age}}}}$$

A Gompertz growth function, adapted from Ricker (1975):

(2) $$D{W_{age}} = {\rm \; }D{W_{birth}} \times {\rm exp}\left( {\ln \left( {\displaystyle{{D{W_{inf}}} \over {D{W_{birth}}}}} \right)\left( {1 - \exp \left( { - k \times age} \right)} \right)} \right)$$

A von Bertalanffy growth function, adapted from von Bertalanffy (1938):

(3) $$D{W_{age}} = D{W_{birth}} + \left( {D{W_{birth}} - D{W_{inf}}} \right) \times \left( {1 - \exp \left( { - k \times age} \right)} \right)$$

These growth functions describe the relationship between age and body size (disc width; DW), with the asymptotic disc width $D{W_{inf}}$, the size at birth $D{W_{Birth}}$, the growth coefficient $k$, the estimated age based on vertebrae growth band counts ( $age)$, and the predicted size-at-age $D{W_{age}}$. Parameters were estimated using Bayesian MCMC models (Bürkner, 2017; Bürkner, 2018).

The prior values for size at birth (10 cm) and maximum disc width (34 cm) are based on data recorded by Moore, Séret & Armstrong (2019) and given a lognormal prior as these were positive parameters. Hence, for the size at birth ( $D{W_{birth}}$) prior a lognormal distribution of 10 and a standard deviation of 1 was used. For the maximum disc width prior a lognormal distribution of 34 and a standard deviation of 1 was used. Lastly, for the growth coefficient ( $k$) a prior with a normal distribution of –1 and a standard deviation of 1 was used. For each model, four chains were run with 3,500 iterations each, including 1,000 discarded warm-up iterations, so a total of 10,000 iterations were sampled for each model. Effective sample sizes for each model parameter exceeded 1,000. Convergence and mixing of chains were monitored with trace plots and R-hat statistics. Model performance was compared using the leave-one-out cross-validation using the ‘loo’ R-package (Vehtari, Gelman & Gabry, 2017; Yao et al., 2018).

### Maturity

We determined the maturity stage as either ‘immature’ or ‘mature’. Females are regarded as mature when epigonal organ is present, ovaries contain very well-developed follicles of similar sizes or are atretic and vitellogenic in groups or singular, and uteri are tubular to wide in shape, with developed walls or with distinguishable embryos. Lastly, males are regarded as mature when little epigonal organ is present, testis have a high volume, are fully lobulated with increased blood supply or pale and decreased in size, and ductus deferens is strongly undulated. Individuals were regarded as immature if reproductive organs were in a less developed state, then described above. Table 1 provides short descriptions used to determine maturity stage. To calculate the median disc width at maturity ( $D{W_{50}}$) for both sexes combined and separated, we used the following logistic maturity formula (Mollet et al., 2000):

(4) $$Y = {(1 + {e^{ - \left( {a + bX} \right)}})^{ - 1}}$$

**Table 1:** Developmental stages of reproductive organs used to assess maturity stage (immature or mature).

Sex		Immature	Mature
**Female**	**Ovaries**	Not distinguishable	Distinguishable
	**Follicles**	Underdeveloped or in groups with different sizes	Well-developed or atretic and vitellogenic
	**Uteri**	Between tubular and wide in shape with developed walls	Tubular to wide in shape, developed walls, possibly with embryos
	**Epigonal organ**	Predominant	Present
**Male**	**Testis**	Lobulated, low blood supply	High volume, lobulated, increased blood supply
	**Ductus deferens**	Barely or not undulated	Strongly undulated
	**Epigonal organ**	Present	Limitedly present

**Note:**

Adapted from Osaer et al. (2015).

Median size at maturity is calculated using Eq. (5) similarly, for this model 3,500 iteration and 1,000 warm-up iterations were used. The priors used were uninformative, namely 10 following a normal distribution with a standard deviation of 5 for both $a$ and $b$ as this could not be based on previous values.

(5) $$D{W_{50}} = - a/b$$

### Diet

Stomachs of sampled specimens were removed and weighted prior to determining stomach contents. Excess moisture was removed from stomach contents using paper towels to remove weight bias by stomach fluids. Stomach contents were sorted into one of six categories: crustaceans, polychaetes, bivalves, other molluscs, teleosts or unidentified (unrecognizable prey items). Figure S1 provides representative photo of each taxa encountered in stomach contents. These taxa categories were not defined prior to data collection but based on prey items encountered due to the lack of description for benthic species from our study area. For each group, we recorded the number of prey items and mass to the nearest centigram. To prevent bias of large prey items, we calculated the diet composition using the index of importance ( $IOI$) as proposed by Gray, Mulligan & Hannah (1997). First, the percentage of each prey group relative to the body weight of the individual ( $\% {W_a}$) was calculated as:

(6) $$\% {W_a} = \left( {100 \times {W_a}} \right)/{W_{body}}$$

where ${W_a}$ is the sum weight of prey group a (Gray, Mulligan & Hannah, 1997). Secondly, the frequency of occurrence for prey group a ( $\% {F_a}$) was calculated as:

(7) $$\% {F_a} = \left( {100 \times {S_a}} \right)/S$$

where ${S_a}$ is the number of stomachs containing for a given prey group, and $S$ denotes the total number of stomachs containing food (Hyslop, 1980). Lastly, the index of importance for each prey group ( $IO{I_a}$) was calculated as:

(8) $$IO{I_a} = 100 \times \; H{I_a}/\mathop \sum \limits_{a = 1}^n HI$$

with:

(9) $$H{I_a} = \% {F_a} + \% {W_a}$$

Diet composition was analyzed for three different life stages: YOY (individuals < 1 year of age), juveniles (individuals >= 1 year, but have not reached $D{W_{50}}$) and adults (mature individuals > $D{W_{50}}$). Raw data is provided in Table S1. We performed a PERMANOVA (R-package ‘vegan’) (Oksanen et al., 2020) and a pairwise Adonis function (Martinez Arbizu, 2016) to determine which life stages differ in their diet composition.

## Results

A total of 75 individual *F. margaritella* were sampled consisting of 38 males and 37 females (0.5:0.5 m:f ratio) ranging from 12.2 to 30.6 cm DW and body mass ranging from 59 to 1,208 g.

### Age and growth

**Figure 2:**
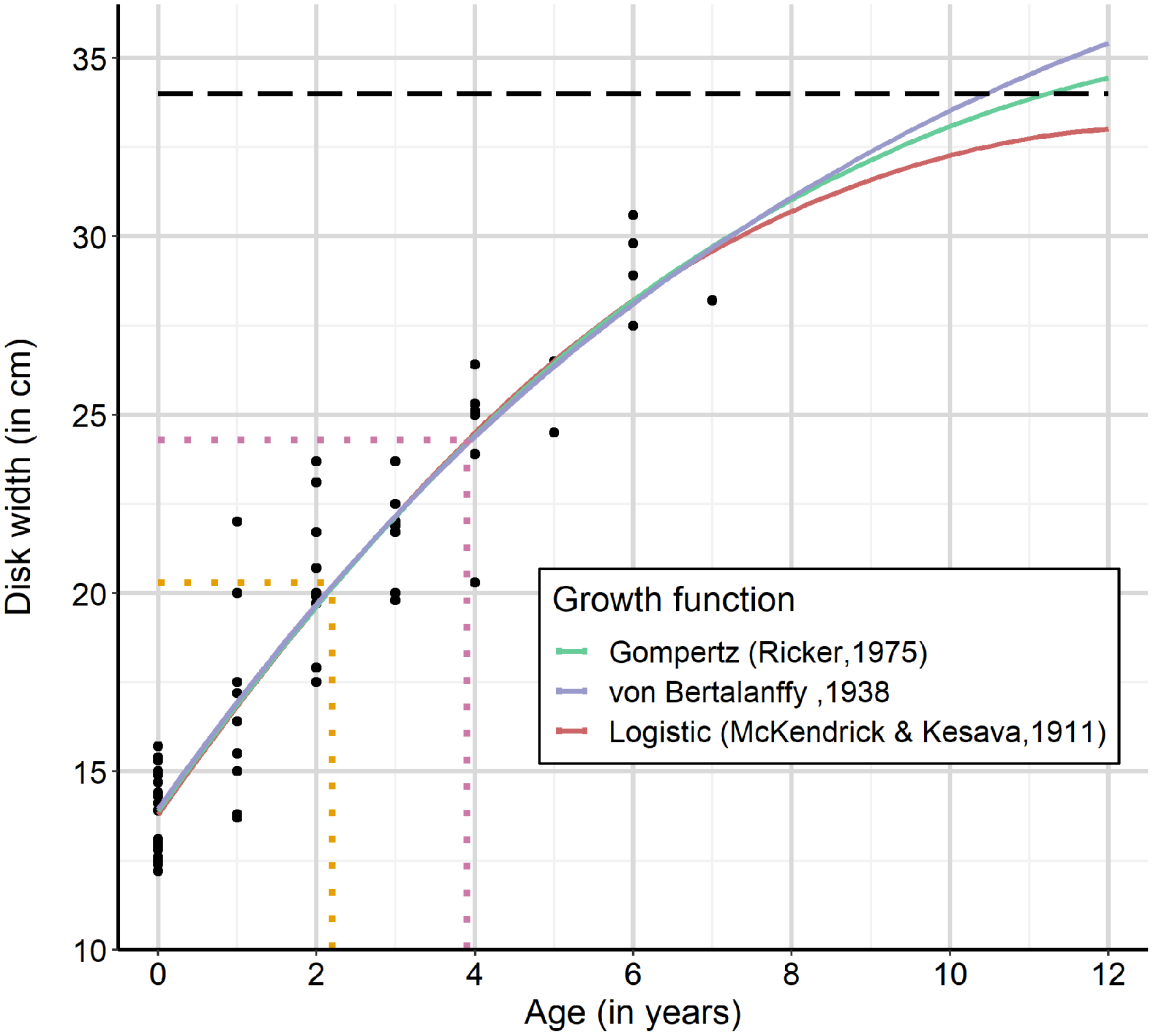
Growth functions fitted to size-at-age data of *F. margaritella* (Gompertz curve in green, von Bertalanffy curve in blue and the logistic growth curve in red). The horizontal dashed line represents the maximum reported disc width of 34 cm (Moore, Séret & Armstrong, 2019). The median disc width at which males reach maturity (DW_50_) is shown in orange (DW = 20.3 cm, age = 2.2 year) and magenta for females (DW = 24.3 cm, age = 3.9 year).

A total of 71 individuals (*m* = 38, *f* = 33) were used for size-at-age analysis. Measured disc widths ranged from 12.2 to 30.6 cm (18.7 ± 5.1 cm), and age ranged from less than 1 to 7 years (1.8 ± 1.9 years). All three growth functions estimated similar values for disc width size-at-birth: 13.87, 14.01 and 14.01 cm (von Bertalanffy, Gompertz and Logistic growth functions respectively). Maximum disc width estimates varied between the three growth functions. The logistic growth function estimated a maximum disc width of 34.46 cm, close to the observed maximum size of 34 cm, recorded by Moore, Séret & Armstrong (2019), whereas the von Bertalanffy function estimated 44.70 cm and the Gompertz function 38.09 cm (Table 2). Model selection showed that no model outperformed any of the others based on LOO information criterion (LOOIC) (Table 2). However, there is likely little reliable difference in the predictive capability between these models, as the difference in LOOIC values was less than two between all models. When considering the maximum reported size by Moore, Séret & Armstrong (2019) to be 34 cm as the maximum disc width, *F. margaritella* individuals seem to reach their maximum size between 10 and 12 years (Figure 2). Additionally, *F. margaritella* seems to attain between 40.3% and 64.7% of their maximum disc width within their first year, based on the largest and smallest rays of one year old.

**Table 2:** Model and model selection estimates for the: the von Bertalanffy, Gompertz and the logistic growth function.

Model	*N*	LOOIC	SE	DW_∞_ (cm)	95% CI DW_∞_	DW_BIRTH_ (cm)		95% CI DW_BIRTH_	K (year)^–1^	95% CI K
Von Bertalanffy function	71	278.7	14.2	44.7	33.1–75.2		13.9	13.3–14.4	0.1	0.04–0.2
Gompertz function	71	279.4	14.8	38.1	30.9–55.1		14.0	13.3–14.6	0.2	0.1–0.3
Logistic function	71	280.1	15.1	34.5	29.4–44.2		14.0	13.5–15.0	0.3	0.2–0.4

**Notes:**

*n*, sample size for size at age analysis; LOOIC, LOO information criterion; SE, standard error of the LOOIC values; DW_∞_, asymptotic disc width; DW_Birth_, disc width at birth; K, growth rate; 95% CI, credible interval.

### Maturity

We determined the maturity stage of 69 individuals (*m* = 35, *f* = 34). Of six individuals the reproductive state was unclear fishery-related damages or (partial) decomposition of organs. The disc width of the largest sampled immature male was 23.7 cm, and the largest immature female had a disc width of 30.6 cm. Based on the binomial logistic maturity regression median size-at-maturity is achieved at 20.3 cm DW (95% CI [18.8–21.8 cm]) for males and 24.3 cm DW (95% CI [21.9–26.5 cm]) for females (see Table 3). Based on the von Bertalanffy growth function, this size-at-maturity corresponds with an age-at-maturity of 2.2 and 3.9 years for males and females, respectively.

**Table 3:** *S*ummary of size-at-maturity parameter estimates and 95% credible interval values for males, females, and both sexes combined.

Sex	a	a–95% CI	b	b–95% CI	DW_50_	DW_50_–95% CI
Male	−19.25	−34.91 to -9.00	0.95	0.44–1.68	20.3	18.8–21.8
Female	−13.64	−25.22 to -6.37	0.56	0.26–1.03	24.3	21.9–26.5
Combined	−10.5	−16.27 to -6.68	0.5	0.3–0.75	21.0	19.7–22.3

### Diet

**Figure 3:**
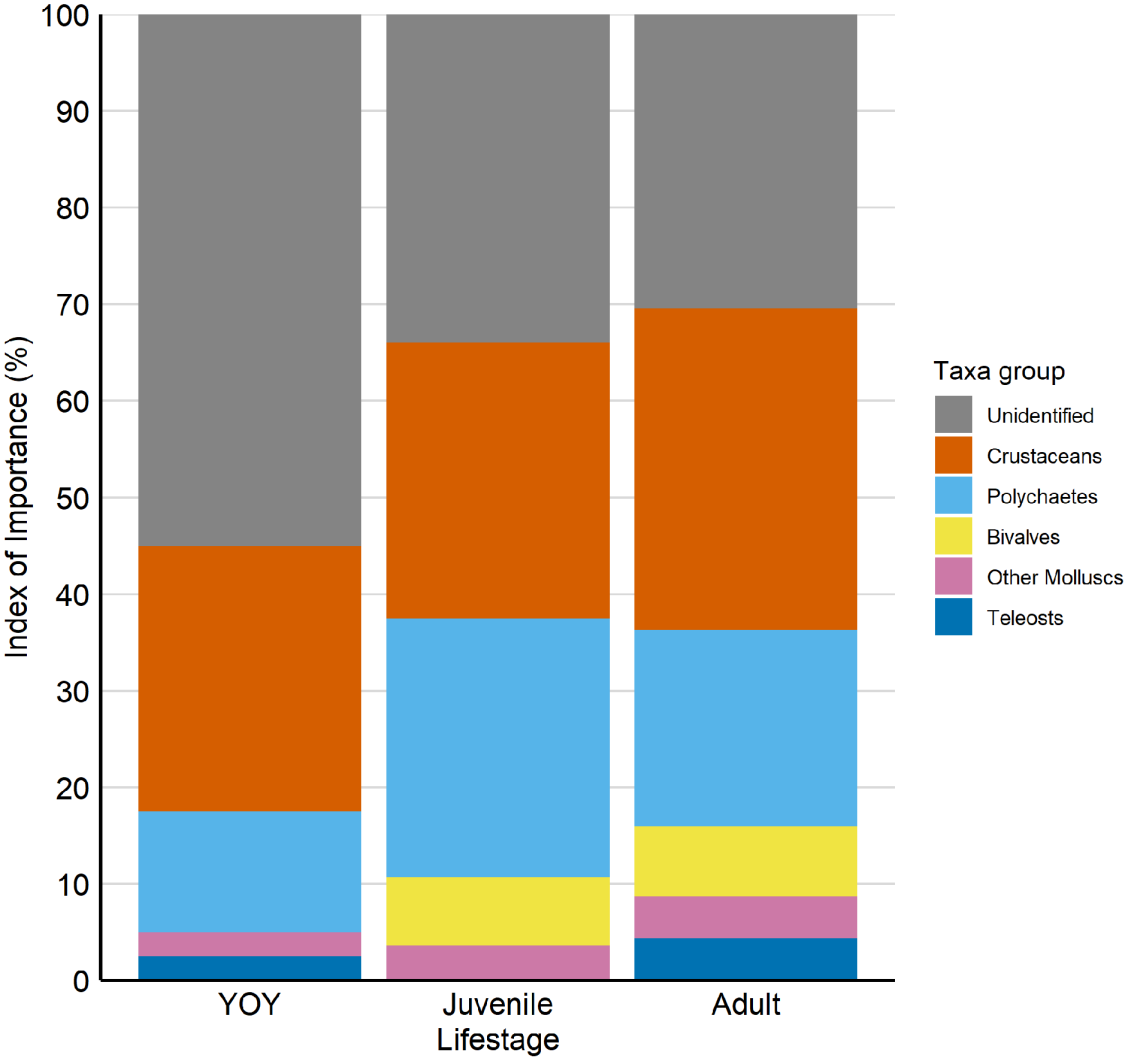
Index of importance (IOI) for each prey taxa for young-of-the-year (YOY), juvenile and adult *Fontitrygon margaritella*. Colours indicate the prey group taxa. Crustaceans (orange). Polychaetes (light blue). Bivalves (yellow). Other Molluscs (violet). Teleost fishes (dark blue). Unidentified (gray). Raw data is provided as Table S1.

For diet analysis we used a total of 65 stomachs were sampled (1.19 ± 1.25 g). We identified 22 individuals as young-of-the-year (YOY; <1 year of age), 19 as juvenile (>= 1 year of age and smaller than DW_50_) and 24 as adults (>= DW_50_). Based on the Index of Importance, the same prey species made up the majority of *F. margaritella’s* diet across all life stages, crustaceans: 27.4–33.3%, polychaetes: 12.5–26.7%, Bivalves: 12.5–20.3, other molluscs: 0–7.2%, Teleosts: 0–4.3%, and unidentified prey: 30.4–55.0% (Figure 3). Besides unidentified prey, crustaceans and Polychaetes were the most common prey items for all age classes both in mass and number of individual prey (Table 4).

**Table 4:** Summary of the total mass (grams), total count (*n*) and percentage of stomachs that contained crustaceans and polychaetes for YOY, Juveniles and adults.

	YOY	Juvenile	Adult
Crustaceans mass (g)	0.8	3.5	17.5
Crustaceans count (*n*)	224	270	331
Nr. stomachs (%)	50	84.2	95.8
Polychaetes mass (g)	1.0	1.4	5.5
Polychaetes count (*n*)	68	99	331
Nr. stomachs (%)	22.7	78.9	58.3

Diet composition differed significantly between life stages (PERMANOVA DF = 2, sum of squares = 2.3, *F* = 22.6, *R*^2^ = 0.27, *p* = 0.001), and a post-hoc test revealed that all life stages have a significantly different diet composition (YOY-Juveniles: *F* model = 7.8, *R*^2^ = 0.1, *p* = 0.002) (YOY-Adults: *F* model = 17.2, *R*^2^ = 0.28, *p* = 0.001) (Juveniles-Adults: *F* model = 6.0, *R*^2^ = 0.1, *p* = 0.001.

## Discussion

Elasmobranchs are still subject to fisheries in coastal waters of west Africa despite their vulnerability to fishing (Moore, Séret & Armstrong, 2019). Understanding the life-history and trophic ecology of elasmobranch species is essential for the risk assessment of both these species and the ecosystems in which they often play a key-role. This study is the first to present detailed data about the growth, median size-at-maturity and the diet of the poorly studied *F. margaritella* in the Bijagós Archipelago, Guinea-Bissau.

Based on the growth curves of *F. margaritella* seems to achieve the maximum recorded disc width size of 34 cm between 10 and 12 years. Surprisingly, the maximum age of our sampled specimens was only 7 years (*n* = 1). One-year old *F. margaritella* are between 40.3% and 64.7% of their maximum disc width, which is comparable to the fast-growing Roger’s stingray (*Urotrygon rogersi*) (Mejía-Falla et al., 2014). In addition, a slow growing batoid species (*Dasyatis fluviorum*) has been observed to have a growth coefficient of around 0.03 year^–1^ (Pierce & Bennett, 2010), which is around a third of the growth coefficient observed for *F. margaritella* of 0.10 year^–1^. The growth rate that we found, is comparable to other fast-growing species such as Rogers stingray (*Urotrygon rogersi*), Kuhl’s maskray (*Neotrygon kuhlii*) and the Diamond stingray which is between 0.1 and 0.24 year^–1^ (Mejía-Falla et al., 2014; Temple et al., 2020).

Our study indicates that in the Bijagós Archipelago male *F. margaritella* mature at an earlier age compared to females. This has also been confirmed in other ray species such as the brown stingray (*Dasyatis lata*) and the common stingray (*Dasyatis pastinaca*) (Ismen, 2003; Dale & Holland, 2012). This sex difference in size-at-maturity can have several possible causes. For instance, this could be related to male biting behaviour during reproduction, which is common in many elasmobranch species (Kajiura, Sebastian & Tricas, 2000). Unlike males, large females of Haller’s round ray (*Urolophus halleri*) have been observed to obtain a relatively thicker disc with increased disc width, which may help minimize damage from male reproductive biting behaviour (Nordell, 1994). Alternatively, larger females are thought to produce larger litters and therefore have a greater reproductive output (Lyons et al., 2017), which could be a reason female *F. margaritella* mature later and at a larger body size. Perhaps a more likely explanation may be that size-at-maturity may also vary based on the increased energetic expenditure during the gestation period (Goodwin, Dulvy & Reynolds, 2002). Females of *F. margaritella* reach maturity at around 32.5% of their lifespan, and males around 18.3% (considering a maximum age of 12 years). This is similar to other species such as the Kuhl’s maskray (*Neotrygon kuhlii)* and the blackspotted whipray (*Maculabatis gerradi)*, which mature between 19% and 41% of their lifespan (Temple et al., 2020). However, whether size-at-maturity differs in other areas remains unknown. Our estimates of male and female median size-at-maturity should be interpreted with caution due to low sample sizes. However, a study on Baraka’s whiprays (Maculabatis ambigua) by Temple et al. (2020) provided an accurate estimate for size-at-maturity for males based on a low sample size. Furthermore, the approximation of male maturity by Last et al. (2016) (~21 cm) differs only 7 mm (0.3%) from our estimation and falls within the range of our (95% CI [18.8–21.8 cm]), supporting our median size-at-maturity estimations for male *F. margaritella*.

Additionally, the gestation period and frequency needs verification to assess reproductive rate of *F. margaritella*, as this is thought to vary within the family of *Dasyatidae* (Carlson et al., 2020; Notarbartolo di Sciara et al., 2015). Hence, to get a well-rounded comprehension of the life-history of *F. margaritella* gestation period and frequency should also be studied.

We found that the diet of *F. margaritella* within the Bijagós Archipelago consisted mostly of crustaceans and polychaetes. This indicates that *F. margaritella* acts as a small, low trophic level meso-predator that links benthos communities with top-predators in the Bijagós Archipelago. The presence of teleost items the stomach contents of *F. margaritella* suggests that the species occasionally consumes teleost prey, as observed in other batoid species (Lim et al., 2019; Farias et al., 2006). Whereas other studies show that batoids ontogenetically include more teleost prey (Gray, Mulligan & Hannah, 1997; Lim et al., 2019; Farias et al., 2006), in our study one YOY was observed to have consumed small teleost prey. The high proportion of unidentified prey encountered likely results from soft-bodied prey (*e.g*., polychaetes and small crustaceans) which may digest faster (Farias et al., 2006). The unidentified prey items could also be inorganic matter, sediment and plant matter ingested during prey consumption (Ajemian & Powers, 2012). DNA metabarcoding on stomach contents could improve estimates of prey abundance and, combined with environmental DNA analysis of benthos, may highlight prey preference (Harms-Tuohy, Schizas & Appeldoorn, 2016). We found that *F. margaritella* undergoes an ontogenetic diet shift and adults seem to incorporate more diverse prey into their diet such as teleosts and a higher abundance of crustaceans, possibly giving older individuals a slightly higher trophic level. Ontogenetic diet shifts could result from changes in teeth morphology, jaw teeth strength, body size and sensory sensitivity of the peripheral (Kempster et al., 2013; Lim et al., 2019; Nordell, 1994; Smith & Merriner, 1985). Ontogenetic diet shifts may also result from different energetic needs as well as local prey availability coinciding with ontogenetic differences in distribution (Lim et al., 2019). Regardless, ontogenetic diet shifts could suggest that different life stages fulfil different trophic roles and affect food webs differently.

Comprehensive knowledge on the life-history and ecology of a species is necessary to establish adequate conservation efforts (Ismen, 2003; O’Shea et al., 2013). With many elasmobranch populations declining globally, the need for insight into their life-history and trophic ecology for conservation increases. This study presents one of the first known estimates for growth, median size-at-maturity, and diet composition of *F. margaritella*. Compared to other ray species *F. margaritella* seems to be a fast growing and early maturing species. The diet description presented in this paper may provide preliminary insights into their trophic role in coastal ecosystems of west-Africa. Additionally, fishing intensity, natural mortality rate and recruitment rate of *F. margaritella* require study to assess fishing vulnerability (Le Quesne & Jennings, 2012). This study contributes to the knowledge on *F. margaritella*, a common exploited elasmobranch species in the West African region, and may help conservation efforts of this, and other exploited elasmobranch species in this region.

## Conclusions

*Fontitrygon margaritella* is a small, relatively fast-growing ray species, reaching maturity after 2.2 and 3.9 years for males and females respectively. The diet of this species within the Bijagos Archipelago consists primarily of polychaetes, but the contribution of harder prey species (*e.g*. crustaceans) increases ontogenetically. This study presents the first description of growth, median size-at-maturity and diet of *F. margaritella*, needed for science-based management of coastal fisheries and coastal ecosystems. These results fill an important knowledge gap on the life history and trophic ecology of this species, and this data-deficient genus of whiprays.

## Supplemental Information

10.7717/peerj.12894/supp-1 Supplemental Information 1 Representative photo of each taxa group found in stomach content. (A) crustaceans, (B) bivalves, (C) teleosts, (D) other mollusks, (E) polychaetes, (F) unidentified.

10.7717/peerj.12894/supp-2 Supplemental Information 2 Life stage and stomach content weights in grams for each specimen.

## Additional Information and Declarations

### Field Study Permissions

The following information was supplied relating to field study approvals (*i.e*., approving body and any reference numbers): Approval for this reseach was given by the Instituto da Biodiversidade e das Áreas Protegidas (IBAP).

### Data Availability

The following information was supplied regarding data availability: The data and the R script used for analysis are available at DataverseNL: Clements, Owen N.; Leurs, Guido; Witbaard, Rob; Pen, Ido; Verkuil, Yvonne I.; Govers, Laura L., 2022, ‘‘Replication Data for: Growth, maturity, and diet of the pearl whipray (*Fontitrygon margaritella*) from the Bijagós Archipelago, Guinea-Bissau’’, https://doi.org/10.34894/BUXZJU, DataverseNL, V1.
