# Supplementary material for: Growth, maturity, and diet of the pearl whipray (Fontitrygon margaritella) from the Bijagós Archipelago, Guinea-Bissau
Source: PeerJ. 2022 Mar 7;10:e12894. doi: 10.7717/peerj.12894 (PMC8908892; doi:10.7717/peerj.12894)
Supplement: Supplemental Information 1 — (A) crustaceans, (B) bivalves, (C) teleosts, (D) other mollusks, (E) polychaetes, (F) unidentified. [file peerj-10-12894-s001.pdf]

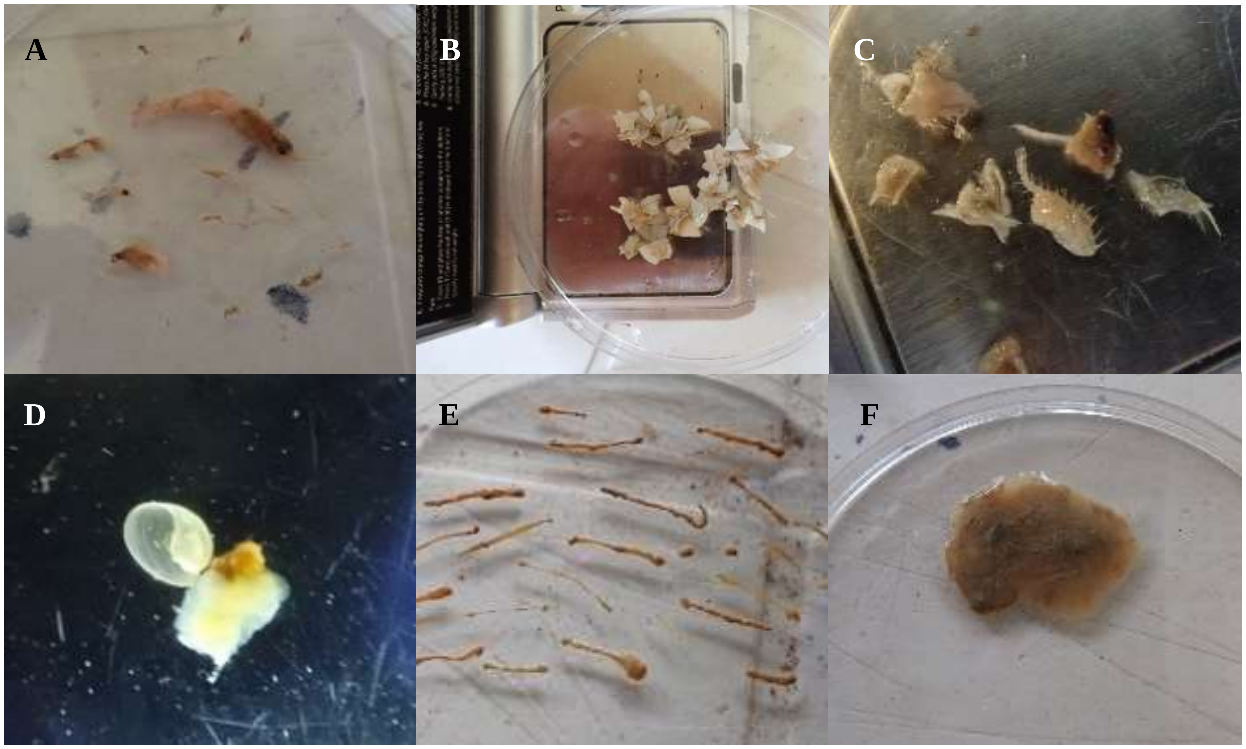

Figure S1: Representative photo of each taxa group found in stomach content. (A) crustaceans. (B) bivalves. (C) teleosts. (D) other molluscs. (E) polychaetes. (F) unidentified.
