# Supplementary material for: Growth, maturity, and diet of the pearl whipray (Fontitrygon margaritella) from the Bijagós Archipelago, Guinea-Bissau
Source: PeerJ. 2022 Mar 7;10:e12894. doi: 10.7717/peerj.12894 (PMC8908892; doi:10.7717/peerj.12894)
Supplement: Supplemental Information 2 [file peerj-10-12894-s002.docx]

**Table S1:** **Life stage and stomach content weights in grams for each specimen.**

| Life stage | Unidentified (g) | Crustacea  (g) | Worm (g) | Bivalve (g) | Mollusk (g) | Teleost (g) | Total content (g) |
| --- | --- | --- | --- | --- | --- | --- | --- |
| Adult | 0.369 | 0.27 | 0.001 | 0 | 0 | 0 | 0.569 |
| Adult | 3.6 | 0.14 | 1.17 | 0 | 0 | 0 | 5.41 |
| Adult | 0.49 | 0.85 | 0.06 | 0 | 0 | 0 | 1.34 |
| Adult | 0.8 | 0.4 | 0.2 | 0 | 0 | 0 | 1.8 |
| Adult | 1.1 | 1.3 | 0.4 | 0 | 0 | 0 | 3.1 |
| Adult | 0.23 | 0.78 | 0.33 | 0 | 0 | 0 | 1.12 |
| Adult | 0.05 | 0.06 | 0 | 0 | 0 | 0 | 0.12 |
| Adult | 0.24 | 0.29 | 1.24 | 0 | 0 | 0 | 2.01 |
| Adult | 1.44 | 2.05 | 0 | 1.75 | 0 | 0 | 6.35 |
| Adult | 0.23 | 0.36 | 0.04 | 0 | 0.02 | 0 | 0.62 |
| Adult | 0.05 | 0.71 | 0.05 | 0 | 0 | 0 | 0.86 |
| Adult | 0.28 | 0.33 | 0.04 | 0 | 0 | 0 | 0.67 |
| Adult | 1.38 | 0.96 | 1.04 | 0 | 0 | 0 | 4.3 |
| Adult | 0 | 0 | 0 | 0 | 2.83 | 0 | 2.83 |
| Adult | 0.229 | 0.001 | 0.21 | 0 | 0.24 | 0 | 0.759 |
| Adult | 0.6 | 2.21 | 0.45 | 0 | 0 | 0 | 3.26 |
| Adult | 0.61 | 0.04 | 0.27 | 0.88 | 0 | 0.83 | 3.239 |
| Adult | 0.76 | 1.12 | 0 | 0 | 0 | 1.9 | 4.34 |
| Adult | 0 | 1.65 | 0 | 0 | 0 | 0 | 1.65 |
| Adult | 0.28 | 1.39 | 0 | 0.41 | 0 | 0 | 1.98 |
| Adult | 0.47 | 0.63 | 0 | 0 | 0 | 0 | 1.1 |
| Adult | 0 | 1.47 | 0 | 0 | 0 | 0 | 1.47 |
| Adult | 0.11 | 0.09 | 0 | 0.04 | 0 | 1.31 | 1.55 |
| Adult | 0.14 | 0.45 | 0 | 0.23 | 0 | 0 | 0.74 |
| Juvenile | 0.5 | 0.16 | 0.11 | 0 | 0 | 0 | 1.19 |
| Juvenile | 0.16 | 0 | 0.15 | 0 | 0 | 0 | 0.18 |
| Juvenile | 1.06 | 0.04 | 0 | 0 | 0 | 0 | 2.09 |
| Juvenile | 0.409 | 0.52 | 0.12 | 0.001 | 0 | 0 | 1.149 |
| Juvenile | 0.11 | 0.03 | 0 | 0 | 0 | 0 | 0.14 |
| Juvenile | 1.21 | 0.26 | 0.08 | 0 | 0.03 | 0 | 1.83 |
| Juvenile | 0.399 | 0.04 | 0.001 | 0 | 0 | 0 | 0.519 |
| Juvenile | 0.888 | 0.76 | 0.001 | 0.001 | 0 | 0 | 2.278 |
| Juvenile | 0.2 | 0.03 | 0.06 | 0 | 0 | 0 | 0.29 |
| Juvenile | 0.21 | 0.67 | 0.12 | 0 | 0 | 0 | 0.9 |
| Juvenile | 0.95 | 0.19 | 0 | 0 | 0 | 0 | 1.18 |
| Juvenile | 1.339 | 0.34 | 0 | 0 | 0.001 | 0 | 1.909 |
| Juvenile | 0.3 | 0.03 | 0.03 | 0 | 0 | 0 | 0.36 |
| Juvenile | 0.589 | 0.13 | 0.001 | 0 | 0 | 0 | 0.979 |
| Juvenile | 0.22 | 0.04 | 0.07 | 0 | 0 | 0 | 0.52 |
| Juvenile | 1.68 | 0 | 0.04 | 0.1 | 0 | 0 | 2.58 |
| Juvenile | 1.02 | 0 | 0.13 | 0 | 0 | 0 | 1.17 |
| Juvenile | 0.029 | 0.28 | 0.05 | 0.001 | 0 | 0 | 0.359 |
| Juvenile | 0.73 | 0.01 | 0.09 | 0 | 0 | 0 | 1.56 |
| YOY | 0.23 | 0.1 | 0.23 | 0 | 0 | 0 | 0.58 |
| YOY | 0.179 | 0.001 | 0 | 0 | 0 | 0 | 0.319 |
| YOY | 0.51 | 0.04 | 0 | 0 | 0 | 0 | 0.56 |
| YOY | 0.26 | 0 | 0 | 0 | 0 | 0 | 0.26 |
| YOY | 0.049 | 0.06 | 0.001 | 0 | 0 | 0 | 0.159 |
| YOY | 1.01 | 0.14 | 0 | 0 | 0 | 0 | 1.19 |
| YOY | 0.16 | 0.05 | 0.15 | 0 | 0 | 0 | 0.38 |
| YOY | 0.28 | 0.17 | 0 | 0 | 0 | 0 | 0.45 |
| YOY | 0.2 | 0 | 0.07 | 0 | 0 | 0 | 0.27 |
| YOY | 0.289 | 0.001 | 0.03 | 0 | 0 | 0 | 0.389 |
| YOY | 0.52 | 0.04 | 0.05 | 0 | 0 | 0 | 0.71 |
| YOY | 0.38 | 0 | 0 | 0 | 0 | 0 | 0.38 |
| YOY | 0.17 | 0.03 | 0 | 0 | 0 | 0 | 0.23 |
| YOY | 0.36 | 0.02 | 0 | 0 | 0 | 0 | 0.39 |
| YOY | 0.139 | 0.001 | 0 | 0 | 0 | 0 | 0.139 |
| YOY | 0.67 | 0.08 | 0.19 | 0 | 0 | 0 | 0.95 |
| YOY | 0.119 | 0 | 0.001 | 0 | 0 | 0 | 0.129 |
| YOY | 0.137 | 0.001 | 0.001 | 0 | 0.001 | 0 | 0.197 |
| YOY | 0.278 | 0.001 | 0.001 | 0 | 0 | 0 | 0.328 |
| YOY | 0.17 | 0.06 | 0 | 0 | 0 | 0.01 | 0.24 |
| YOY | 0.098 | 0.001 | 0.001 | 0 | 0 | 0 | 0.148 |
| YOY | 0.24 | 0.04 | 0 | 0 | 0 | 0 | 0.28 |
